# Rare Case of Leiomyoma and Adenomyosis in Mayer-Rokitansky-Kuster-Hauser Syndrome

**DOI:** 10.1155/2016/3725043

**Published:** 2016-10-23

**Authors:** P. S. Hoo, A. R. Norhaslinda, J. N. Shah Reza

**Affiliations:** Obstetrics & Gynaecology Department, School of Medical Science, Universiti Sains Malaysia, Kubang Kerian, Kelantan, Malaysia

## Abstract

We report a case of adenomyosis which developed from a hypoplastic uterus and leiomyoma in a patient with MRKH syndrome. A 45-year-old Malay female with primary amenorrhoea and primary infertility presented with abdominal mass and abdominal pain. She is phenotypically female, has well developed secondary sexual characteristics, and has normal female external genitalia with shallow vagina dimple. Transabdominal ultrasonography showed a homogenous adnexal mass of 10 × 8 cm, uterus sized 5 × 4 cm, and normal kidneys. A complex mass of right adnexa was demonstrated by CT scan. Exploratory laparotomy showed torsion of right adnexal mass and rudimentary uterus with fibroid but no endometrial tissue and blind end with absent cervix. The normal right ovary and tube were not visualized. The left fallopian tube and ovary were normal. It is also complicated by vaginal agenesis. Removal of right adnexal mass and rudimentary uterus was done with preservation of left ovary. The histologic diagnosis was uterine adenomyosis and leiomyoma arising from the right adnexa, possibly from the broad ligament.

## 1. Introduction

Mullerian agenesis, a congenital malformation of the genital tract, is a common cause of primary amenorrhea, second only to gonadal dysgenesis [1]. It affects one in 4,000–5,000 female births. The most common presentation of mullerian agenesis is congenital absence of the vagina, uterus, or both, which also is referred to as mullerian aplasia or vaginal agenesis or Mayer-Rokitansky-Kuster-Hauser syndrome. Its aetiology is poorly understood and it may be associated with renal, skeletal, cardiac, and auditory anomalies. The diagnosis is often made either radiologically or laparoscopically in amenorrhoea women with normal hormonal tests and female karyotype.

## 2. Case Presentation

A 45-year-old Malay female, with primary amenorrhoea and primary infertility for 11 years, presented with abdominal mass and abdominal pain. She had sought treatment for her primary amenorrhoea when she was 16 years old, with no further investigation after then. She got married at the age of 34 years. There was no complaint of difficulty during sexual intercourse with her partner. Investigations were done for primary infertility after one year of marriage and diagnostic laparoscopy was offered but patient refused.

She presented with abdominal mass for one year, which gradually increased in size and associated with intermittent pain. There was no bowel or bladder compressive symptoms.

She is phenotypically female, with normal intelligence and average height. No hirsutism or acne was noted. Examination of the head and neck did not reveal a webbed neck or any abnormal facies. Thyroid was not enlarged. There were no gross abnormalities of the extremities such as polydactyly, syndactyly, or absence of digit. Chest and heart examinations were normal. There was no complaint of galactorrhoea.

On abdominal examination, a nontender, fixed suprapubic mass with measurement of 15 by 15 cm was palpable with regular margin and firm consistency. There were no ascites or hepatosplenomegaly. The breasts and pubic hair were in Tanner stage 4 with normal female external genitalia. Vagina examination revealed shallow vaginal canal with length of 2 cm and cervix was not palpable.

**Figure 1:**
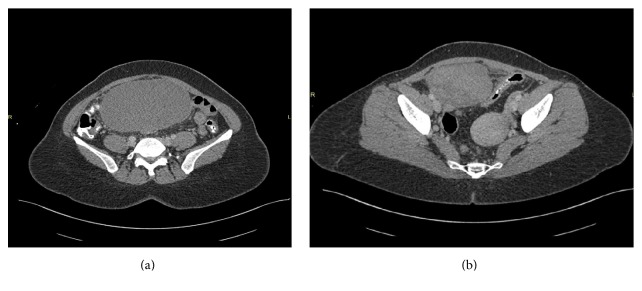
MRI.

Transabdominal ultrasonography showed a homogenous adnexal mass of 10 × 8 cm. The uterus was visualized measuring about 5 × 4 cm. The kidneys were in normal in site, size, and position. MRI revealed a complex mass of the right adnexal measuring 8.8 × 13.8 × 15.2 cm as shown in Figures 1(a), 1(b), and 4. Uterus is retroverted; left adnexa and ovary are within normal size. Kidneys are normal.

Gonadotropins level were normal: Follicle stimulating hormone (FSH) was 5.5 IU/L and luteinizing hormone (LH) was 5.1 IU/L. Serum level of CA-125 was 226 U/mL. Provisional diagnosis of MRKH syndrome associated with pelvic mass (leiomyoma or ovarian tumour) was made and exploratory laparotomy was offered.

**Figure 2:**
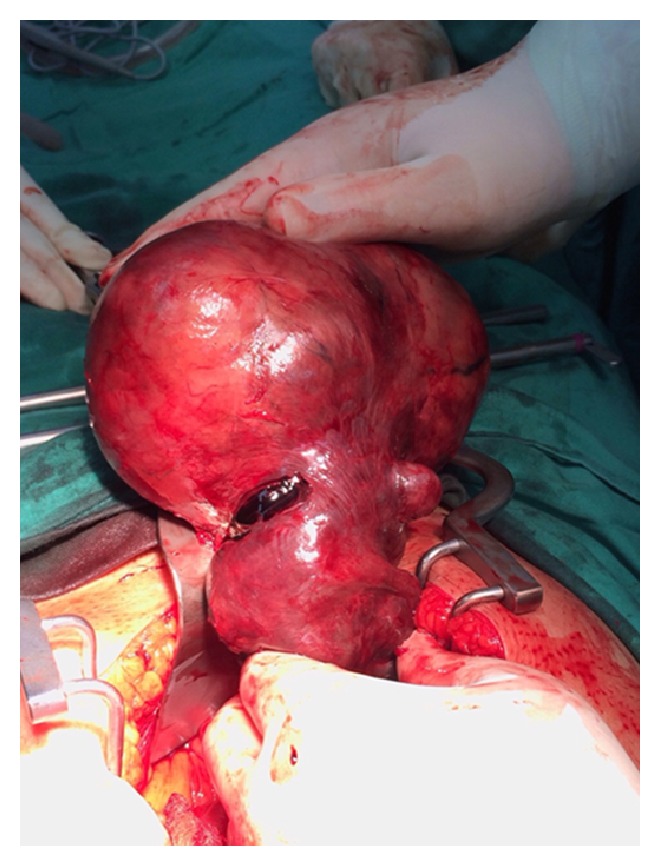
Twisted right adnexal mass.

**Figure 3:**
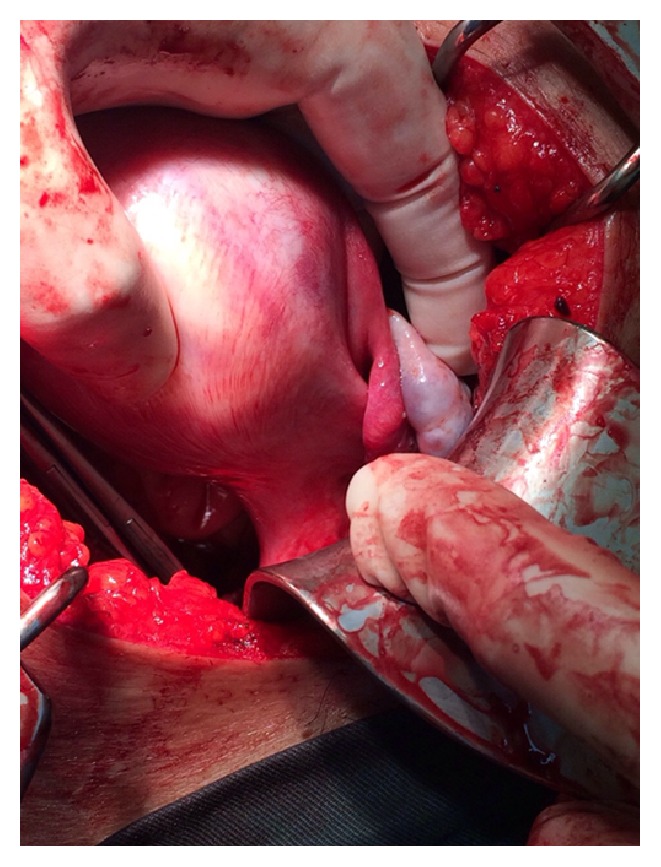
Enlarged left horn of the uterus. Normal left ovary and left fallopian tube.

**Figure 4:**
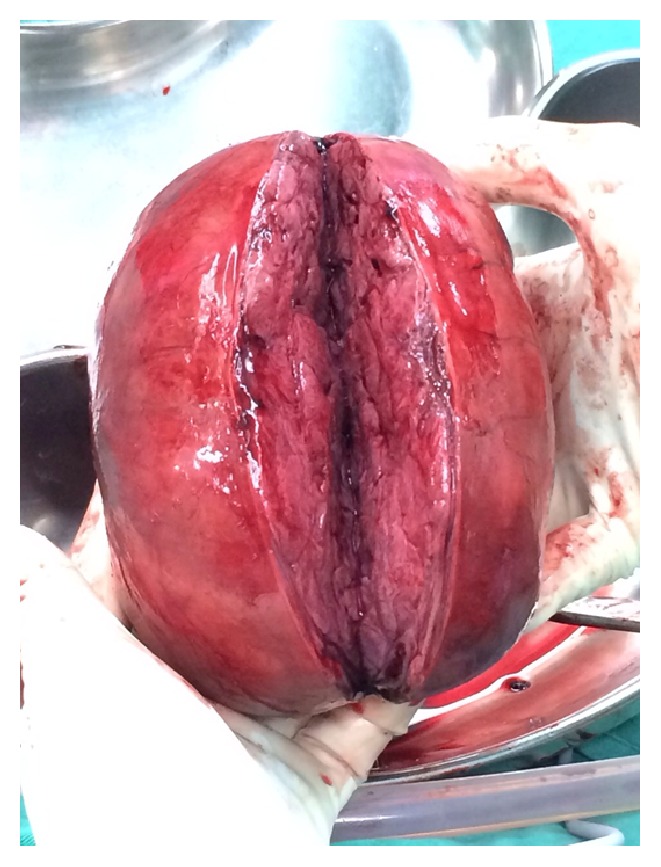
Cut section of right adnexal mass.

**Figure 5:**
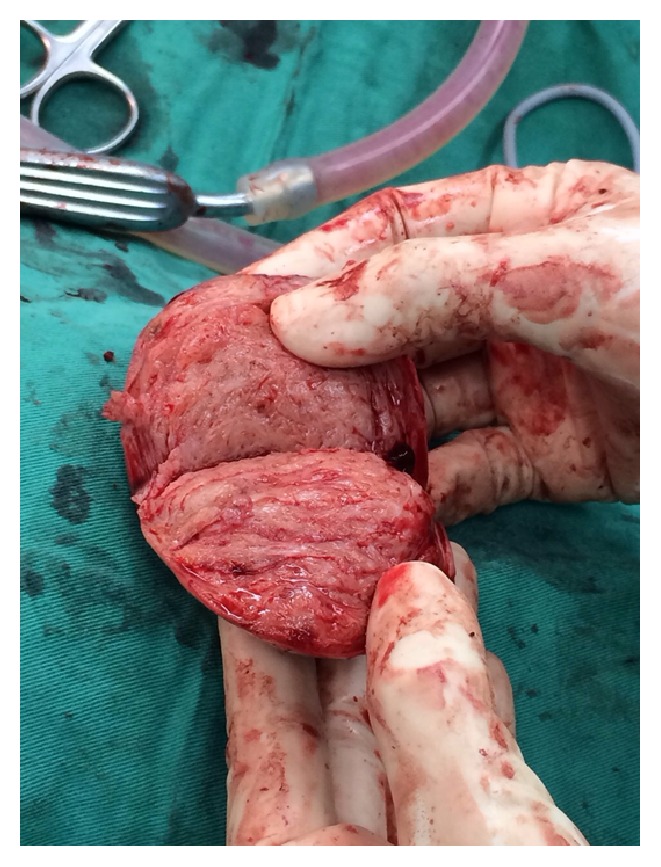
Cut section of enlarged left horn of the uterus.

Intraoperatively, torsion of right adnexal mass of 15 × 13 × 13 cm was found, freely mobile, together with enlarged left horn of the uterus measuring 5 × 5 cm which has no endometrial tissue, blind end with no cervix (Figures 3 and 5). There were no abnormal or enlarged vessels noted. The right ovary and tube were not visualized. The left fallopian tube and ovary were normal. It is also complicated by vaginal agenesis. Removal of the twisted right adnexal mass and rudimentary uterus was done with preservation of left ovary (Figure 2).

The patient was offered for neovaginoplasty, but she refused. Postoperative recovery was uneventful.

Histopathological examination was reported as leiomyoma arising from the right adnexae and structure was possibly broad ligament. While the enlarged left rudimentary uterus showed adenomyosis which involved part of the uterus, right ovary and right fallopian tube showed haemorrhagic necrosis possibly secondary to torsion.

## 3. Discussion

MRKH syndrome is a rare disorder described as aplasia or hypoplasia of uterus and upper two-thirds of vagina due to early arrest in development of mullerian duct. Women with this syndrome have normal 46 XX karyotype, secondary sex characters, ovarian functions, and underdeveloped vagina [2]. Renal, skeletal, hearing, and cardiac anomalies may be an association. Presence of leiomyoma and adenomyosis in MRKH syndrome is very rare and only few cases have been reported in the literature.

Here, we report a patient of MRKH syndrome with a large leiomyoma originating from the broad ligament and adenomyosis from the rudimentary uterus. Leiomyomas of uterus are oestrogen-dependent tumours. Although mullerian ducts are primarily endodermal in origin, some smooth muscle cells may exist at their proximal ends, which may be the origin of leiomyomas. However, the exact etiopathogenesis of leiomyoma from the rudimentary uterus in MRKH syndrome is not known. Parikh stated that fibroids and adenomyosis rarely develop in the rudimentary nonfunctioning uterus [3]. The first case of adenomyosis in MRKH syndrome was reported by Enatsu et al. [4]. Yan and Mok reported a case of 52-year-old Chinese woman with MRKH syndrome, who underwent hysterectomy and bilateral salpingo-oophorectomy for painful uterine mass and was diagnosed with uterine fibroids and adenomyosis [5]. Noncommunicating uterine horns of unicornuate uterus have also been reported to have myomas [6].

Conventionally uterine adenomyosis always represents a downgrowth from the basal layer of the endometrium, which means that adenomyosis arises through direct invasion of the uterine mucosa into the uterine musculature [7]. But this theory failed to explain how adenomyosis develops in mullerian remnants in patient with MRKH syndrome. This case, with the absence of a functional endometrial lining, does not support the abovementioned theory. Another hypothesis promotes the possibility that metaplasia of the stromal cells under the influence of autocrine factors or paracrine factors which are the intermediaries of genetic, immunologic, and endocrine influences can lead to the development of adenomyosis in situ. Therefore, the histogenesis of adenomyosis in our case may not be a result of direct invasion but due to metaplasia of mullerian remnants inside the hypoplastic uterus. Enatsu et al. reported that there existed endometrium-like tissues in myometrium of their patient who did not have functional endometrium [4]. A case of simple endometrial hyperplasia of ectopic endometrial tissue in myometrium with normal endometrial cavity in patient with adenomyosis was reported by Chun et al. [8]. The authors stated the possibility of spontaneous hyperplasia of ectopic endometrium independent of eutopic endometrium, which partially supports the hypothesis of metaplasia in the development of adenomyosis.

## 4. Conclusion

Women with MRKH syndrome who present with abdominal mass and pain, endometriotic ovarian cysts, adenomyosis, or leiomyoma of mullerian remnant should be considered for diagnosis. Ultrasonography is the first modality to evaluate intra-abdominal masses and genitourinary system. CT and MRI are more accurate modalities to delineate the intra-abdominal masses before planning for surgery. Complete removal of the masses with the uterine remnant by either laparotomy or laparoscopy is recommended.
